# Mood and Risk-Taking as Momentum for Creativity

**DOI:** 10.3389/fpsyg.2020.610562

**Published:** 2021-01-21

**Authors:** Tsutomu Harada

**Affiliations:** Graduate School of Business Administration, Kobe University, Kobe, Japan

**Keywords:** mood, risk-taking, divergent and convergent thinking, representational change theory, Q learning model

## Abstract

This study examined the effects of mood and risk-taking on divergent and convergent thinking using a Q-learning computation model. The results revealed that while mood was not significantly related to divergent or convergent thinking (as creative thinking types), risk-taking exerted positive effects on divergent thinking in the face of negative rewards. The results were consistent with the representational change theory in insight problem solving. Although this theory accounts directly for insight, the underlying idea of going beyond current contexts and implicit constrains could be applied to creative thinking as well. The results indeed accounted for the relevance of this theory to divergent thinking. The current study is one of the first empirical studies simultaneously examining the role of mood and risk-taking in creativity. In particular, no related studies exist that took a computational approach to estimate the relevant parameters in the framework of dynamic optimization. Our Q learning model enables to distinguish and identify the different roles of mood and risk-taking in updating *Q* values and making decisions.

## Introduction

Creativity inevitably requires learning. Although learning can proceed in a logical and consistent manner, as suggested by reinforcement learning (RL) models, it also relies on mood. According to decision affect theory, mood is affected by unexpected outcomes or reward prediction errors (RPE), which represent the difference between the actual reward and the expected reward in the RL framework (Mellers et al., 1997; Shepperd and McNulty, 2002). As a result, subsequent learning performance is either promoted or obstructed (Eldar and Niv, 2015).

Mood has been extensively studied as a predictor of creativity (Isen and Baron, 1991; Mumford, 2003). This is because mood often serves as “an intermediary state between a host of situational and personality predictors, on the one hand, and creative performance, on the other” (Baas et al., 2008). In the mood-creativity literature, while a number of studies have emphasized the importance of positive mood in creative thinking (for example, Ashby et al., 1999; Lyubomirsky et al., 2005), several exceptions exist that showed that a positive mood sometimes leads to less creativity (Kaufmann and Vosburg, 1997; Anderson and Pratarelli, 1999). Moreover, some studies showed that a negative mood improves creative performance.

Given these contradictory findings, Baas et al. (2008) conducted a meta-analysis and reported that “positive moods produce more creativity than mood-neutral controls (*r* = 0.15), but no significant differences between negative moods and mood-neutral controls (*r* = 0.03) or between positive and negative moods (*r* = 0.04) were observed.” According to their analysis, creativity seems to be facilitated by “positive mood states that are activating in nature and associated with an approach motivation and promotion focus (e.g., happiness), rather than those that are deactivating and associated with an avoidance motivation and prevention focus (e.g., relaxed)” (Baas et al., 2008).

As another candidate for the determinant of creativity, risk-taking attitudes have been extensively studied because creative persons are more likely to be motivated by challenging and risky situations (Albert, 1990; Perkins, 1990), suggesting that risk-taking is closed related to creativity. While the theoretical significance of the relationship between risk-taking and creativity has been recognized (Eisenman, 1987; Sternberg and Lubart, 1992; Feist et al., 1998; Dewett, 2007), only a few empirical studies have examined the relationship. Most of these empirical studies reported that creativity and risk-taking were positively correlated (Eisenman, 1987; El-Murad and West, 2003; Dewett, 2007; Simmons and Ren, 2009; Tyagi et al., 2017; Harada, 2020a). However, Shen et al. (2018) found that low risk-taking was associated with convergent thinking, but risk-taking was not significantly correlated with divergent thinking. Probably, diversity in the research measures, definitions of risk-taking, and cultural backgrounds of the participants in the different studies accounted for these differences in results (Strum, 1971).

Despite these inconclusive results, both positive mood and risk-taking serve to relax and break implicit constraints that hinder problem solving and creative thinking. According to representational change theory (Ohlsson, 1992; Knöblich et al., 1999), insight problem solving initially involves the construction of an erroneous problem space. Representational change takes place through the relaxation of constraints such as the abandonment of unnecessarily constraining assumptions. Positive mood and risk-taking attitudes provide a strong impetus for challenging the existing rules of the game to remove unnecessary constraints and create more appropriate problem spaces. Taken together, we hypothesize that divergent thinking is facilitated by positive mood and risk-taking because new insights, as critical ingredients of divergent thinking, are considered to be a function of cognitive flexibility, which is enabled by the removal of underlying constraints.

To examine this hypothesis, we took a computation approach to estimate mood and risk-taking attitudes. In the current study, we measured mood using a model proposed by Eldar and Niv (2015), and examined its effect on creativity. Creativity is defined as a combined manifestation of novelty and usefulness (Sternberg and Lubart, 1999; Jung et al., 2010) and has often been identified with divergent thinking. Divergent thinking is defined as the ability to generate multiple solutions to an open-ended problem (Guilford, 1967). Thus, divergent thinking reflects the notion that creativity is more likely to proceed in an unpredictable and abrupt manner. In addition to divergent thinking, convergent thinking has also been highlighted as a factor accounting for creativity (Abraham, 2018). Convergent thinking, which is the ability to apply conventional decision-making strategies to produce an already known answer (Cropley, 2006), is sometimes instrumental in generating insight problem solving (Bowden et al., 2005). Accordingly, related studies seem to support that creativity research should take into account both divergent and convergent thinking (Gabora, 2010). Thus, we examined the effects of mood and emotional state on divergent and convergent thinking, in addition to exploitation and exploration.

With mood and risk being the determinants of creativity, the mood literature (Isen and Baron, 1991; Mumford, 2003) primarily examined the effects of risk attitudes, whereas the risk literature (Eisenman, 1987; El-Murad and West, 2003; Dewett, 2007; Simmons and Ren, 2009; Tyagi et al., 2017; Shen et al., 2018; Harada, 2020a) evaluated the influences of mood without reference to mood effects. None of the literature considered the simultaneous effects of risk attitudes and mood on creativity. This study could be differentiated from related prior studies in that we tested the effects of both positive mood and risk-taking on creativity using a rigorous computational approach.

Thus, our empirical analysis made explicit the underlying computational model of mood and risk-taking, upon which relevant parameter estimates were derived. We sought to test the effects of mood and risk-taking on divergent and convergent thinking.

## Materials and Methods

This study used the data analyzed in Harada (2020a, b) with permission, but the model and estimation adopted in this study differed from the latter in that the effects of mood were incorporated.

### Participants

Our experiments were announced in one of the undergraduate courses the author taught at Kobe University, and some undergraduate students applied voluntarily. A total of 127 participants took part in the experiments, but 14 of them were excluded from the final sample because they did not attend one of the two sessions. As a result, the sample of this study consisted of data collected from 113 healthy undergraduate students of Kobe University (49 females, age range = 18–20 years, SD = 0.66). All participants were native Japanese-speakers with normal or corrected-to-normal vision. The local Ethics Committee approved this study. All participants signed an informed consent form before taking part in the experiment, and were paid JPY 3000 (approximately USD 28).

### Procedure

The participants completed the S-A creativity test, Remote Associates Test (RAT), reading span, operation span, and matrix span tests (Conway et al., 2005), the Iowa Gambling Task (IGT), and the Big Five Scales (BFS) for personality traits. The experiments were arranged into two independent sessions: An S-A session (including S-A creativity test and RAT) and an IGT session (including reading span, operation span, matrix span tests, the IGT, and the BFS). To remove the order effects on test scores, approximately half of the participants performed the S-A session first and then the IGT session while the remaining participants performed the sessions in the opposite order. There was at least a 7-day interval between the two successive sessions.

During the S-A session, participants completed both the S-A test and the RAT, each of which took approximately 30 min. During this session, the tests were completed in accordance with the instructor’s manuals. A break of at least 5 min was taken between the two tests. To remove order effects, the order of the tests was randomly assigned.

The IGT session was arranged in groups with a maximum of 20 participants who completed the tests in the presence of an instructor. The tests were performed on a 17” CRT monitor with PsyToolkit (Stoet, 2010, 2017). A break of at least 1 min was given between every two tests. The order of the tests was randomly assigned in PsyToolkit across the participants to exclude the order effects on test scores. In the IGT, the participants were instructed to maximize the total sum of rewards. Additionally, they were informed that some of the decks might generate higher expected rewards. No other information was provided regarding the IGT and the test took approximately 30 min to be completed. Each of the reading span, operation span, and matrix span tests took approximately 5 min, and the BFS took 15 min. Thus, it took approximately 60 min to complete all of the tests in the IGT session.

### Q Learning Model

This study adopted a RL framework to account for behavior in evaluating options for decision-making in the IGT. The RL framework has been applied to the study of multi-armed bandit problems and is supported by a number of empirical evidences including neural signals in various cortical and subcortical structures that behaved as predicted (Schultz et al., 1997; Glimcher and Rustichini, 2004; Hikosaka et al., 2006; Rangel et al., 2008). The framework has also been applied to studies on decision making and learning in various social contexts (Delgado et al., 2005; Montague et al., 2006; Behrens et al., 2008; Hampton et al., 2008; Coricelli and Nagel, 2009; Bhatt et al., 2010; Yoshida et al., 2010). However, little attention has been paid to the creative aspects of decision making.

To measure mood and risk-taking attitude observed in decision making in the IGT, we used a variant of the Q learning model in the RL framework (Sutton and Barto, 2018). The participants make a series of 100 choices from 4 decks of cards. Two of the decks are advantageous and the other two are disadvantageous. The two disadvantageous decks always yield relatively high gains ($100), but also occasional large losses ($150) with a 50% chance, resulting in an average loss of $25 per trial. The two advantageous decks always generate lower gains ($50) but produce no losses ($0) with a 50% chance, resulting in an average gain of $25 per trial. The goal is to maximize net scores across trials.

At each trial t, the action value *Q*_*i*_(*t*) of the chosen option (deck) i is updated via the following rule:

(1) $${\text{Q}}_{i}\,\left(t+1\right)=\left\{ \begin{array}{c} Q_{i}\,\left(t\right)+\alpha^{+}\,\delta \,\left(t\right)+\phi \,i\,f\,\delta \,\left(t\right)\geq 0,\\ Q_{i}\,\left(t\right)+\alpha^{-}\,\delta \,\left(t\right)+\phi \,i\,f\,\delta \,\left(t\right)<0, \end{array}\right.$$

with,

(2) $$\delta \,\left(t\right)=U\,\left(R_{i}\,\left(t\right)\right)\,f^{m}-{\text{Q}}_{i}\,\left(t\right),$$

(3) $$U\,\left(R_{i}\,\left(t\right)\right)=\left\{ \begin{array}{c} R_{i}\,{\left(t\right)}^{\mu }\\ -\lambda \,{\left(-R_{i}\,\left(t\right)\right)}^{\nu }, \end{array}\right.$$

where *R*_*i*_(*t*) is the reward associated with option i at trial t, and α^±^ indicates the learning rate. ϕ is added as the choice trace to account for autocorrelation of choice, which can affect learning biases (Katahira, 2018). *U*(*R*_*i*_(*t*)) takes the form of the prospect utility function proposed by Tversky and Kahneman (1986) in which μ and ν measure the degrees of risk aversion and risk seeking, respectively. We adopted this utility function because one of our research interests was to examine the effect of risk attitudes on creativity. Thus, it was assumed that participants would evaluate the reward in terms of their own risk attitudes, which resulted in the utility function specified in (3). δ(*t*) represents the RPE. The RPE is computed by subtracting the current value estimate from the obtained reward R. Participants thus update the action value estimate by scaling the prediction error with the learning rate, then adding this to the estimated value in the previous trial. Learning rates close to 1 indicate that a person performs fast adaptation based on prediction errors, and learning rates closer to 0 indicate slow adaptation. In the default setting, the initial action values were set to zero so that *Q*_*i*_(1) = 0i = 1,…,4.

As described in Eldar and Niv (2015), f is a positive constant that represents the mood bias and m is the participants’ mood. If f = 1, mood is neutral without biasing the perception of rewards. If f > 1, positive feedback exists such that positive and negative mood are magnified, while f < 1 indicates negative feedback, stabilizing the effect of mood over time. Mood (m) is specified to reflect the prediction error history (h) as:

(4) $$\text{h}\,\left(t+1\right)=\text{h}\,\left(t\right)+\zeta_{h}\,\left(\delta \,\left(t\right)-\text{h}\,\left(t\right)\right),$$

Given this prediction history, mood is defined as a sigmoid function of h :

(5) m⁢(t)=tanh⁡(h⁢(t)),

This implies that m takes values between [−1,  1]. Thus, m indicates good (0 < m < 1) or bad (−1 < m < 0) mood. Good and bad moods, respectively, increase and decrease *Q* values of current choices. According to Eldar and Niv (2015), the mood inferred from this model accords with participants’ self-reported feeling throughout their experiment. Thus, we assumed m and f captured the mood and its biases in our experiment as well.

For the unselected option j (i≠j), the action value is updated as:

(6) $$Q_{j}\,\left(t+1\right)=Q_{j}\,\left(t\right)$$

We assume that the chosen action at trial t is denoted by a(*t*) ∈ {1, 2, 3, 4}. The action value estimates of these four options are used to determine the probability of choosing either option. This probability is computed via the following softmax decision rule:

(7) $$P\left(a\left(t\right)=i\right)=\frac{e\,x\,p\,\left(\beta \,Q_{i}\,\left(t\right)\right)}{\sum_{j=1}^{4}e\,x\,p\,\left(\beta \,Q_{j}\,\left(t\right)\right)}$$

where *P*(*a*(*t*) = *i*) is the probability of choosing the action *a*(*t*) = *i* at trial t. The parameter β is the inverse temperature, a parameter that indicates the sensitivity of a participant’s choice to the difference in action value estimates.

The parameters of αt± and β in this model were estimated by optimizing the maximum a posteriori (MAP) objective function, to find the posterior mode:

(8) $$\hat{\theta }=a\,r\,g\,m\,a\,x\,p\,\left(D_{s}|\theta_{s}\right)\,\text{p}\,\left(\theta_{s}\right)$$

where *p*(*D*_*s*_|θ_*s*_) is the likelihood of data *D*_*s*_ for subject s conditional on parameters θ_*s*_ = {^α±*S*^,μ^*S*^,ν^*S*^,λ^*S*^,ϕ^*S*^,^*fS*^,ζ^*S*^,β^*S*^}, and p(θ_*s*_) is the prior probability of θ_*s*_. We assumed that each parameter is bounded and used constrained optimization to find the MAP estimates. Specifically, since α^±^ is bounded between 0 and 1, and μ,ν,λ,β and f take non-negative values, their priors were assumed to follow beta distributions for α^±^, and gamma distributions for μ,ν,λ,β*and*f. Given these parameter estimates, we calculated the average m values over 100 trials in the IGT for each participant.

### Measures

In the current study, divergent and convergent thinking were used as dependent variables. We focused on examining the effects of mood (m) and risk-taking measures (μ*and*ν) on divergent and convergent thinking scores. Working memory capacity and personality characteristics, which might affect the dependent variables, were used as control variables.

#### Divergent Thinking

Divergent thinking is defined as the ability to produce new approaches and original ideas by forming unexpected combinations from available information, and by applying such abilities as semantic flexibility, and fluency of association, ideation, and transformation (Guilford, 1967). In the current study, divergent thinking ability was measured with the S-A creativity test (Society for Creative Minds, 1969), a timed laboratory test corresponding to the measures used in the Torrance Tests of Creative Thinking. The test involves three types of tasks. In the first task, the participants are instructed to generate unique ways of using two objects specified in the test. The second task requires the participants to imagine desirable functions of two specified ordinary objects. In the third task, the participants are instructed to imagine the consequences of “unimaginable things” happening. Each task requires the participants to generate as many answers as possible (up to 10).

The S-A creativity test measures divergent thinking in terms of (a) fluency, (b) flexibility, (c) originality, and (d) elaboration. Fluency is measured by the number of relevant responses to the questions, and is related to the ability to produce and consider many alternatives. Flexibility is the ability to produce responses from a broad perspective, and is measured by the sum of the total number of category types to which answers are assigned based on a criteria table or an almost equivalent judgment. Originality is the ability to produce ideas that differ from others and is scored as the sum of idea categories that are weighted based on a criteria table or an almost equivalent judgment. Elaboration is the ability to produce ideas in detail and is measured by the sum of answers that are weighted based on a criteria table or an almost equivalent judgment. This test also provides a total score for divergent thinking, which was mainly used in this study. For more details about the S-A creativity test, see Takeuchi et al. (2010).

#### Convergent Thinking

Convergent thinking is defined as the ability to apply conventional and logical search, recognition, and decision-making strategies to stored information to produce an already known answer (Cropley, 2006). Thus, convergent thinking requires prior knowledge and is typically correlated with measures of crystallized intelligence. However, most creativity researchers have described convergent thinking as a process entailing the evaluation of initial ideas and/or a sudden insight in arriving at the correct solution for problems with task constraints (Cropley, 2006; Smith and Ward, 2012; Lee and Therriault, 2013). As a result, in the insight problem-solving literature, convergent thinking has typically been measured using the RAT; Mednick, 1962) which entails the task constraint that the correct solution must fit with each of the three words in the presented triad (e.g., “cheese” as the correct response for the triad “cottage, cream, and blue”). As all the participants in this study were native Japanese-speakers, we adopted the Japanese version of the RAT developed by Terai et al. (2013), and used the 40 problems selected by Orita et al. (2018) in our experiment. RAT (convergent thinking) scores were measured by the number of correct solutions for the 40 problems.

#### Mood

In this model, mood can be measured by the magnitude of m in (3). Positive mood is reflected as higher values in m as the exponent of f because it magnifies the utility of choosing the current deck. This means that positive mood tends to reinforce the current choices in the future as well, which implies that an increase in the corresponding utility should be reflected. On the other hand, negative mood discourages repeatedly selecting the current choice in the future, which turns out to be a decrease in the corresponding utility of the current choice. Thus, lower values of m indicate negative mood. As the base of m, f measures the stability of mood.

#### Risk Attitudes

Risk attitudes can be measured by the parameters μ*and*ν in (3), which incorporate part of the prospect utility function in which an asymmetric form of risk aversion is specified. Risk aversion in cases with positive rewards and risk-taking in cases with negative rewards are, respectively, measured by μ*and*ν, indicating that the participants have different risk attitudes toward gains and losses. We were interested in examining these effects on creativity performance.

#### Inverse Temperature

We used the inverse temperature β to represent levels of exploitation and exploration. Exploitation refers to the optimization of current tasks under existing information and memory conditions, while exploration implies wider and sometimes random search and trials that do not coincide with the optimal solutions provided by exploitation (see Sutton and Barto, 2018, for the trade-off between exploitation and exploration in the RL framework), for the trade-off between exploitation and exploration in the RL framework). A higher β value implies that the participants selected the decks based on the action value Q calculated in (1)∼(3), leading to exploitation. Conversely, as β approaches zero, the choice is more likely to have been made randomly because the weight of the *Q* value in the soft max decision rule in (3) significantly declines. This implies that participants undertake exploration. Thus, the inverse temperature β measures the relative importance of exploitation and exploration.

#### Working Memory Capacity (WMC)

Working memory capacity was measured using reading span, operation span, and matrix span tests, which are representative working memory tests (Conway et al., 2005). Reading span and operation span tests evaluate the capacity of verbal WMC and logical WMC, respectively, which in turn correspond to the phonological loop, according to Baddeley (2000). The matrix span test measures spatial WMC, corresponding to the visuo-spatial sketchpad in Baddeley’s model.

#### Big Five Scales of Personality

The BFS of personality traits is widely used to describe personality differences, consisting of five factors: openness to experience (inventive/curious vs. consistent/cautious), conscientiousness (efficient/organized vs. easy-going/careless), extraversion (outgoing/energetic vs. solitary/reserved), agreeableness (friendly/compassionate vs. challenging/detached), and neuroticism (sensitive/nervous vs. secure/confident) (Barrick and Mount, 1991; Miller, 1991; Piedmont et al., 1991). In the current study, these scales were administered using 60 questions in Japanese, developed by Wada (1996). Higher scores on a trait implied that the participant was low on that particular trait. For example, a high score on openness to experience implied lower openness to experience.

The descriptive statistics for all the variables used in the empirical analyses in this study are reported in Table 1.

**TABLE 1:** Descriptive statistics.

	Mean	SD	1	2	3	4	5	6	7	8	9	10	11	12	13
1. Divergent thinking	39.87	10.07	**–**												
2. Convergent thinking	14.49	3.94	–0.08	**–**											
3. Exploitation	40.01	14.31	0.22***	0.09	**–**										
4. Exploration	19.04	7.56	–0.08	–0.13	−0.72***	**–**									
5. Mood	0.01	0	0.04	−0.21**	–0.06	0.01	**–**								
6. Mood bias	0.51	0.3	–0.07	0.05	–0.03	0.11	–0.14	**–**							
7. Extraversion	3.61	0.95	–0.05	0.12	–0.01	0.10	−0.21**	–0.06	**–**						
8. Neuroticism	3.29	0.97	0.08	0.02	–0.05	0.00	0.09	0.04	−0.40***	**–**					
9. Openness	3.89	0.83	−0.16*	0.00	–0.07	0.07	−0.21**	–0.14	0.23***	−0.21**	**–**				
10. Conscientiousness	4.19	0.69	0.00	0.20**	–0.03	–0.12	–0.01	−0.16**	0.08	0.10	0.04	**–**			
11. Agreeableness	3.4	0.89	0.12	–0.08	–0.11	0.14	0.02	–0.01	0.37***	−0.24***	0.14	0.29***	**–**		
12. Spatial WMC	23.81	13.38	0.13	0.16*	0.10	0.09	0.16*	–0.02	0.04	0.00	–0.10	0.01	0.02	**–**	
13. Verbal WMC	25.73	12.75	0.00	0.07	0.03	0.16*	0.02	0.00	0.05	–0.07	–0.02	0.01	–0.08	0.37***	**–**
14. Logical WMC	28.18	11.65	0.05	–0.12	0.04	0.08	0.11	0.03	–0.02	0.01	0.07	–0.01	–0.02	0.22***	0.24***

*p < 0.10, **p < 0.05, ***p < 0.01.

## Results

### Effects of Mood

To examine the effects of mood on divergent and convergent thinking, we regressed mood (m) on divergent and convergent thinking scores with WMC and personality as control variables. The results are shown in Table 2.

**TABLE 2:** Effects of mood (SE in parentheses).

Variables	DT	CT
	(1)	(2)
constant terms	40.14***	8.33**
	(6.41)	(3.86)
Exploitation/Exploration	0.65***	0.14
	(0.18)	(0.11)
Mood	0.69	–1.84
	(2.13)	(1.29)
Mood bias	–103.19	49.16
	(221.84)	(138.13)
Extraversion	–1.11	0.65
	(0.74)	(0.44)
Neuroticism	0.95	0.27
	(0.71)	(0.43)
Openness	−1.98***	0.06
	(0.75)	(0.46)
Conscientiousness	–1.10	1.44***
	(0.92)	(0.55)
Agreeableness	2.42***	−0.90*
	(0.77)	(0.46)
Spatial WMC	0.11**	0.06*
	(0.05)	(0.03)
Verbal WMC	–0.04	0.01
	(0.05)	(0.03)
Logical WMC	0.05	−0.06*
	(0.05)	(0.03)
AIC	899.95	632.84

DT and CT refer to divergent and convergent thinking, respectively. *p < 0.10, **p < 0.05, ***p < 0.01.

The dependent variables in the left and right columns are divergent and convergent thinking scores, respectively. As shown in the table, both mood and mood biases exhibited no significant effect on divergent and convergent thinking. Instead, inverse temperature had a positive effect on divergent thinking. This suggests that exploitation accounts for divergent thinking.

Regarding the control variables, noting that high scores on the personality scales meant low trait presence, we found that agreeableness exerted a negative effect on divergent thinking, whereas openness to experience and spatial WMC exerted positive effects on divergent thinking. Agreeableness is amenable to conservative behavior, implying that highly divergent thinkers tend to break conservative behavior with its negative effects. Moreover, openness to experience indicates curiosity about new experiences, which is also expected to promote divergent thinking. These results are consistent with the representational change theory, that is, highly divergent thinkers are more likely to challenge and change implicit constraints and problem spaces in problem solving.

In convergent thinking, conscientiousness and spatial and logical WMC exhibited positive effects. Conscientiousness reflects efficient, organized, and thus more careful attitudes, which are instrumental for generating attentive actions. Its negative effects on convergent thinking imply that in the RAT experiment, more exploratory search is required in order to hit upon candidates for correct solutions. Probably, after gaining these candidates, more careful attitudes are required. This phase of convergent thinking might be reflected in the positive effect of agreeableness on convergent thinking, leading to more conservative behavior. Moreover, since our RAT experiment used hieroglyphic Chinese characters, it seems reasonable that spatial and logical WMC had positive effects on convergent thinking.

### Effects of Risk-Taking

Next, we examined the effects of risk-taking on creativity. The results are shown in Table 3.

**TABLE 3:** Effects of risk-taking (SE in parentheses).

Variables	DT	CT
	(1)	(2)
Constant terms	37.51***	8.10**
	(6.02)	(3.59)
Exploitation/Exploration	0.66***	0.13
	(0.18)	(0.11)
H (risk aversion in gains)	3.55	1.18
	(2.17)	(1.31)
v (risk-seeking in losses)	4.03**	0.60
	(2.04)	(1.23)
Extraversion	−1.40*	0.62
	(0.75)	(0.45)
Neuroticism	0.94	0.18
	(0.70)	(0.43)
Openness	−2.17***	0.02
	(0.76)	(0.46)
Conscientiousness	–1.10	1.26**
	(0.92)	(0.55)
Agreeableness	2.47***	−0.92**
	(0.77)	(0.46)
Spatial WMC	0.14***	0.07**
	(0.05)	(0.03)
Verbal WMC	–0.06	0.00
	(0.05)	(0.03)
Logical WMC	0.04	−0.06*
	(0.05)	(0.03)
AIC	894.20	634.18

*p < 0.10, **p < 0.05, ***p < 0.01.

As in Table 2, dependent variables in the left and right columns are divergent and convergent thinking, respectively. In this regression analysis, risk parameters, μ*and*ν were added, instead of the mood parameters. According to the table, the risk-taking index ν was positively associated with divergent thinking. Thus, participants behaved in a risk-taking manner in the face of losses. In contrast, in convergent thinking, risk parameters exhibited no effect, indicating that risk attitudes did not account for performance in convergent thinking. Other parameters showed the same results as shown in Table 2.

### Simultaneous Effects of Mood and Risk-Taking

Finally, we simultaneously regressed mood and risk parameters on divergent and convergent thinking (Table 4). The results regarding the effects of mood, risk attitudes, and control variables remained the same as those in the individual regression analyses (Tables 2, 3), indicating that the results were statistically robust with respect to the regressors. Therefore, our empirical analyses revealed that risk-taking behavior in the face of losses accounted for high divergent thinking, while mood parameters, regardless of positive or negative, exerted no effects on divergent, and convergent thinking.

**TABLE 4:** Simultaneous effects of mood and risk-taking (SE in parentheses).

Variables	DT	CT
	(1)	(2)
Constant terms	38.71***	8.27**
	(6.45)	(3.88)
Exploitation/Exploration	0.64***	0.13
	(0.18)	(0.11)
Mood	0.43	–1.87
	(2.14)	(1.29)
Mood bias	–133.40	42.91
	(222.80)	(138.79)
μ (risk aversion in gains)	3.59	1.21
	(2.18)	(1.31)
ν (risk-seeking in losses)	4.04**	0.54
	(2.04)	(1.23)
Extraversion	−1.39*	0.58
	(0.75)	(0.45)
Neuroticism	0.89	0.26
	(0.71)	(0.43)
Openness	−2.20***	–0.01
	(0.76)	(0.46)
Conscientiousness	–1.17	1.37**
	(0.93)	(0.55)
Agreeableness	2.47***	−0.87*
	(0.77)	(0.46)
Spatial WMC	0.14***	0.06**
	(0.05)	(0.03)
Verbal WMC	–0.06	0.01
	(0.05)	(0.03)
Logical WMC	0.05	−0.07**
	(0.05)	(0.03)
AIC	897.77	635.83

DT and CT refer to divergent and convergent thinking, respectively. *p < 0.10, **p < 0.05, ***p < 0.01.

## Discussion

In this study, we tested the effects of positive mood and risk-taking on creativity using a rigorous computational approach. We found that risk-taking behavior in the face of losses exhibited positive effects on divergent thinking, whereas mood did not play a role in both divergent and convergent thinking. While most of the mood and risk literatures, respectively, emphasized the importance of positive mood and risk attitude as the determinants of creativity, the simultaneous evaluation revealed the contrasting effects between the two variables.

One of the unique contributions of this study lies in the use of measures for risk attitudes and mood. While the related literature primarily adopted the measures evaluated in relevant psychological tests, this study estimated these measures through behavioral characteristics observed in the IGT. Risk attitude was evaluated by the estimates of risk parameters in the underlying utility function (3) and mood was calculated in the model specified in (5). However, this approach has a limitation because alternative measures for risk and mood were excluded in this specification. In particular, the results regarding mood must be interpreted with caution. Mood in this study refers to the fact that participants reinforce the *Q* value of current choices, which is assumed to be caused by their mood. Although this measure was found to be significantly associated with self-reported mood in Eldar and Niv (2015), positive mood under this measure exhibited magnifying effects on *Q* values of current choices. These effects encourage the status quo, and discourage shifting to different choices. Positive mood in this study refers to self-reinforcing forces to increase the *Q* values of current choices. This implies that positive mood impedes challenging and changing current contexts and implicit assumptions. In contrast, positive mood in the related literature encourages challenging and changing current situations and implicit constraints, which corresponded to negative mood in this study. Although our results did not show negative effects of mood, this suggests that positive mood in our model did not at least facilitate divergent thinking.

In addition, although this study was conducted with a relatively large sample, different results could be found in different samples, in particular, in different cultural contexts. For example, Shen et al. (2018) examined the effects of risk-taking on convergent thinking in China. Their results were in contrast to our findings, showing that risk-taking was negatively associated with convergent thinking, but it had no effect on divergent thinking. On the other hand, our results are in line with Harada (2020a), indicating the positive association between risk-taking and divergent thinking and no relationship between risk-taking and convergent thinking. The different results might be caused by cultural differences. As Shen et al. (2018) noted, on the one hand, a positive correlation between divergent and convergent thinking was identified in their study. On the other hand, the study conducted in the Netherlands revealed that these correlations were close to zero or negative (e.g., Chermahini and Hommel, 2010). In this study, the correlation was not statistically significant (see Table 1). Thus, the different results with respect to risk-taking might be attributed to cultural differences between China and Japan.

Despite these limitations, our findings deserve some attention because previous literature examined the effect of either mood or risk-taking on creative thinking, but did not evaluate both at the same time. First, the result that risk-taking attitudes accounted for high scores in divergent thinking is consistent with prior findings and the underlying representational change theory. Divergent thinking requires challenging and changing current contexts and constraints. In particular, in the case of IGT, facing losses several times implies that participants choose high risk, high return decks. In order to maximize the rewards, they have to shift to low risk, low return decks, which seems to be enabled by risk-taking behavior in the face of losses. The results suggest that participants with these risk attitudes tend to obtain high scores in divergent thinking. This is because these participants are more likely to challenge and change their current contexts and constraints in divergent thinking, leading to high scores.

Second, while the current study explicitly defined and measured the magnitude of mood, related studies did not necessarily measure the mood in a parametrically clear manner. Typically, positive mood was induced in participants by giving a small gift such as a package of candy (see, for example, Estrada et al., 1994). However, it remains ambiguous whether the mood variables measured by these methods correspond to self-reinforcing or self-destructive forces with respect to current contexts and implicit assumptions. The positive moods induced by some gifts are too broad to apply to the specific decision-making situations such as the IGT. Since our measure for mood derived from actual behaviors in the IGT, it seems more relevant to account for learning behaviors of participants.

Third, the positive effects of inverse temperature on divergent thinking deserve to be mentioned here. As described above, inverse temperature measures the relative levels of exploitation and exploration. Noting that exploitation measures the optimization under existing information, its positive effect on divergent thinking may seem to contradict the representational change theory. This is because exploitation seems to reinforce current choices, rather than shifting to different choices. However, the inverse temperature in this model incorporated risk-taking forces because *Q* values in (1) include the risk attitudes specified in (3). Consequently, the positive effect of the inverse temperature on divergent thinking facilitates, rather than impedes, risk-taking attitude reflected in ν. Thus, this result is indeed consistent with the positive effect of risk-taking on divergent thinking.

Forth, the results revealed that neither risk-taking nor inverse temperature accounted for convergent thinking performance. However, caution should be exercised when interpreting this result because the RAT required both divergent and convergent thinking to yield correct solutions. Moreover, the correct solution was sometimes obtained through insight. A combination of problem solving with and without insight makes it difficult to identify the relative contributions of risk-taking and exploitation/exploration ratio. As described above, insight is caused by the representational change, which requires some explorative activities. Problem solving without insight, however, proceeds in an incremental manner. Consequently, it is possible that the mixing of problem solving with and without insight in the RAT caused ambiguity regarding the effects of risk-taking and exploitation/exploration ratio.

By and large, the results of this study are consistent with the representational change theory. According to this theory, problem solving initially involves the construction of an erroneous problem space, making it infeasible to come up with correct solutions. Representational change can then occur through constraint relaxation. This relaxation is enabled by automatic and unconscious processes. Smith and Kounios (1996) and Topolinski and Reber (2010) emphasized the interplay between conscious and unconscious mechanisms in problem solving, and provided a framework for interpreting insight as the conscious correlate of processing fluency caused by a sudden appearance of the solution. Although there exists a debate regarding whether insight occurs through a sudden or gradual process, most researchers share the view that insight, as one of the most important ingredients of creative thinking, is enabled by representational change. This change is facilitated by risk-taking and self-destructive mood. The finding that risk-taking is positively related to divergent thinking, but self-reinforcing mood does not account for either divergent or convergent thinking is consistent with the representational change theory.

However, our study differs from the typical representational theory in that the underlying decision-making process is explicitly modeled. The representational change in our model is caused by shifting to seemingly non-optimal choices. This shift is enabled by increasing the *Q* value of such choices. Thus, any parameters that induce this increase lead to the representational choice. In our model, they correspond to risk-taking parameters. The inverse temperature, as a proxy for exploitation/exploration ratio, could also account for explorative behavior. However, as noted above, since risk-taking behavior was reflected in the *Q* values, exploitation, rather than exploration, played a significant role in divergent thinking. One of the advantages of this computational approach to the representational change is that we could interpret and discuss precisely how each parameter effect is related to the representational change in terms of increasing non-optimal choices. Related studies in the representational change theory, without exception, hinge on verbal and conceptual, instead of mathematically rigorous, models. The main disadvantage of verbal and conceptual models is the difficulty of interpreting complex interplays across many relevant variables. As the computational model explicitly models these interactions, this interplay is easily interpreted. Admittedly, model misspecification is a disadvantage of the mathematical model. However, this potential problem should be overcome by proposing more realistic mathematical models, instead of abandoning the computational approach.

On the whole, the results in this study are consistent with the representational change theory, and thus, seem to be generalizable in different cultural contexts. Indeed, Quartiroli et al. (2018) demonstrated the cross-cultural generalizability of the mood profiles (combinations) between English-speaking and Italian-speaking participants. Since this study also examined the effects of mood, their findings support the generalizability of our results. It should be noted, however, that similar mood profiles do not necessarily lead to similar effects on behavior and performance. For example, Ozer (2015) and Giorgi et al. (2020) studied cross-cultural adjustment of expatriate employees and international university students, respectively, attesting its importance in improving performance and the positive role of social support in facilitating adjustment. This implies that cross-cultural adjustment matters in behavioral patterns and performance. Therefore, while the results of this study seem generalizable, it would be difficult to deny the possibility that the relations between mood, risk-taking, and creativity, to some extent, depend on underlying cultural contexts, as demonstrated by Shen et al. (2018). The study of cultural effects on creativity constitutes one of the important future research challenges.

## Conclusion

The current results revealed that risk-taking played a role in providing momentum for exploratory behavior, which in turn facilitated divergent thinking. In contrast, mood as a self-reinforcing force specified in this study was related to neither divergent nor convergent thinking. These results were consistent with the representational change theory in insight problem solving. Although this theory accounts directly for insight, and not necessarily for creative thinking, the underlying idea of going beyond current contexts and implicit constrains could be applied to creative thinking as well. The results indeed account for the relevance of this theory to divergent thinking.

To the best of our knowledge, the current study is one of the first empirical studies simultaneously examining the role of mood and risk-taking in creativity. In particular, no related studies exist that took a computational approach to estimate the relevant parameters in the framework of dynamic optimization. Although mathematical models inevitably entail the risk of misspecification, they have the capacity to clarify interactions across relevant parameters. This allows for estimating the interdependence of relevant parameters in a statistically consistent manner. For example, concepts of exploration, risk-taking, and positive mood are closely related and overlapping so that their mutual effects are usually difficult to evaluate without model specifications. Our Q learning models enable distinguishing and identifying their different roles in updating *Q* values and making decisions. We strongly believe that this computational approach should be applied not only to creativity research but also to other psychological research fields to elucidate underlying cognitive and psychological mechanisms.

Further research is required to explore the role of risk-taking and mood in facilitating or impeding creativity in more detail. In particular, more direct measurements of mood that facilitate exploratory behavior will be required to examine the effects of positive mood on creativity. This constitutes one of our future research challenges.

## Data Availability Statement

The raw data supporting the conclusions of this article will be made available by the authors, without undue reservation.

## Ethics Statement

The studies involving human participants were reviewed and approved by Ethical Committee, Graduate School of Business Administration, Kobe University. The patients/participants provided their written informed consent to participate in this study.

## Author Contributions

The author confirms being the sole contributor of this work and has approved it for publication.

## Conflict of Interest

The author declares that the research was conducted in the absence of any commercial or financial relationships that could be construed as a potential conflict of interest.
